# Redesign and Validation of a Real-Time RT-PCR to Improve Surveillance for Avian Influenza Viruses of the H9 Subtype

**DOI:** 10.3390/v14061263

**Published:** 2022-06-10

**Authors:** Valentina Panzarin, Sabrina Marciano, Andrea Fortin, Irene Brian, Valeria D’Amico, Federica Gobbo, Francesco Bonfante, Elisa Palumbo, Yoshihiro Sakoda, Kien Trung Le, Duc-Huy Chu, Ismaila Shittu, Clement Meseko, Abdoul Malick Haido, Theophilus Odoom, Mame Nahé Diouf, Fidélia Djegui, Mieke Steensels, Calogero Terregino, Isabella Monne

**Affiliations:** 1 EU/OIE/National Reference Laboratory for Avian Influenza and Newcastle Disease, FAO Reference Centre for Animal Influenza and Newcastle Disease, Division of Comparative Biomedical Sciences, Istituto Zooprofilattico Sperimentale delle Venezie (IZSVe), 35020 Legnaro, Italy; smarciano@izsvenezie.it (S.M.); afortin@izsvenezie.it (A.F.); ibrian@izsvenezie.it (I.B.); vdamico@izsvenezie.it (V.D.); fgobbo@izsvenezie.it (F.G.); fbonfante@izsvenezie.it (F.B.); epalumbo@izsvenezie.it (E.P.); cterregino@izsvenezie.it (C.T.); imonne@izsvenezie.it (I.M.); 2 OIE Reference Laboratory for Avian Influenza, Faculty of Veterinary Medicine, Hokkaido University, Sapporo 060-0818, Japan; sakoda@vetmed.hokudai.ac.jp (Y.S.); letrungkiench16@gmail.com (K.T.L.); 3 Department of Animal Health, Ministry of Agriculture and Rural Development (MARD), Hanoi 115-19, Vietnam; chuduchuy@gmail.com; 4 Regional Laboratory for Animal Influenzas and Other Transboundary Animal Diseases, National Veterinary Research Institute (NVRI), Vom 930010, Nigeria; ismaila.shittu@gmail.com (I.S.); cameseko@yahoo.com (C.M.); 5 Laboratoire Central de l’Élevage (LABOCEL), Ministère de l’Agriculture et de l’Elevage, Niamey 485, Niger; haido.malick@yahoo.fr; 6 Accra Veterinary Laboratory, Veterinary Services Directorate, Ministry of Food & Agriculture, Accra M161, Ghana; theodoom@yahoo.com; 7 Laboratoire National de l’Élevage et de Recherches Vétérinaires (LNERV) de l’Institut Sénégalais de Recherches Agricoles (ISRA), Dakar-Hann 2057, Senegal; mamenahe.diouf@isra.sn; 8 Laboratoire de Diagnostic Vétérinaire et de Sérosurveillance (LADISERO), Parakou 23, Benin; djegui_fidelia@yahoo.fr; 9 AI/ND National Reference Laboratory, Sciensano, 1050 Brussels, Belgium; mieke.steensels@sciensano.be

**Keywords:** avian influenza, H9Nx, molecular diagnosis, real-time RT-PCR, validation

## Abstract

Avian influenza viruses of the H9 subtype cause significant losses to poultry production in endemic regions of Asia, Africa and the Middle East and pose a risk to human health. The availability of reliable and updated diagnostic tools for H9 surveillance is thus paramount to ensure the prompt identification of this subtype. The genetic variability of H9 represents a challenge for molecular-based diagnostic methods and was the cause for suboptimal detection and false negatives during routine diagnostic monitoring. Starting from a dataset of sequences related to viruses of different origins and clades (Y439, Y280, G1), a bioinformatics workflow was optimized to extract relevant sequence data preparatory for oligonucleotides design. Analytical and diagnostic performances were assessed according to the OIE standards. To facilitate assay deployment, amplification conditions were optimized with different nucleic extraction systems and amplification kits. Performance of the new real-time RT-PCR was also evaluated in comparison to existing H9-detection methods, highlighting a significant improvement of sensitivity and inclusivity, in particular for G1 viruses. Data obtained suggest that the new assay has the potential to be employed under different settings and geographic areas for a sensitive detection of H9 viruses.

## 1. Introduction

The low pathogenicity avian influenza viruses (LPAIV) of the H9 subtype were described for the first time in the United States of America (Wisconsin, 1966) [1]. After sporadic detections in Eurasia and North America, since the mid-1990s H9 viruses have expanded their geographic range to large territories of Asia, the Middle East and Africa [2,3,4,5] becoming endemic in domestic Galliformes [6,7,8,9,10,11,12]. The dissemination of H9 viruses in different continents resulted in the establishment of two distinct phylogeographic lineages: the American and the Eurasian. The latter gave origin to three main clades, i.e., Y439, Y280 (syn. BJ94) and G1, which includes G1-Western and G1-Eastern subclades [4,5]. Although the H9 hemagglutinin (HA) has been found associated with any of the nine neuraminidase (NA) subtypes, a noticeable preference for the N2 has been observed for the majority of the records [5].

In galliform poultry species, H9N2-induced clinical signs include diminished food and water intake, mild-to-severe respiratory syndromes and a drop in oviposition [13,14,15,16,17,18,19,20,21,22]. High morbidity and mortality can be observed upon secondary or concomitant infections [23,24,25,26,27,28,29]. Importantly, H9N2 viruses of the G1 and Y280 clades showed the ability to also infect mammals and humans as dead-end hosts [30]. Moreover, the cocirculation of zoonotic H9N2 with other AIV subtypes favors the occurrence of genetic reassortment and the emergence of novel viruses of public health concern, as demonstrated by the notorious cases of human infections caused by H5N1, H7N9 and H10N8 harboring the internal gene cassette donated by poultry-adapted H9N2 viruses [30,31,32,33,34,35,36,37,38].

Although H9N2 are non-notifiable, the economic damages and the public health risk posed by these viruses in endemic areas have prompted several countries to undertake vaccination campaigns [15,39,40]. However, the inability of inactivated vaccines to efficiently suppress shedding, suboptimal immunity levels and coverage in flocks, as well as a high heterogeneity in the immunization regimens across poultry production systems, are the most likely causes for increasing antigenic drift and genetic variability in endemic regions [41,42,43].

Because of the important implications on animal and human health, and the impact of vaccination and viral spread on genetic diversification, the development of universal tools for targeted H9 surveillance has clearly become a crucial task. So far, several real-time RT-PCR (rRT-PCR) assays have been developed to identify H9 viruses in birds and/or humans. However, most of these methods appear suboptimal for the universal recognition of all H9 clades, because they were validated with a sample collection under-representing the overall genetic variability of H9, or designed to detect only locally spread variants [44,45,46,47]. Monne and collaborators [48] have developed and validated one of the most widely used protocols for H9 detection that so far has been successfully applied in different contexts and geographic areas [27,49,50,51]. More recently, surveillance activities carried out at the Istituto Zooprofilattico Sperimentale delle Venezie (IZSVe) by employing this method revealed suboptimal detection of G1 strains from Africa and the Middle East, yielding flat amplification plots or false negatives. A subsequent NGS analysis has shown the occurrence of critical mismatches at the probe binding region, most likely being the cause for the observed diagnostic failures. In this study, we performed an in-depth bioinformatics analysis to redesign the assay originally developed by Monne et al., in order to detect H9Nx viruses of any of the Y439, Y280 and G1 clades. The new rRT-PCR was extensively validated and optimized with different reagents to facilitate its acquisition by third-party laboratories. Diagnostic sensitivity was also compared with existing broad-spectrum H9 assays successfully employed for the detection of this subtype [29,48,52]. The newly developed pan-H9 rRT-PCR showed good performances with all the clades and types of matrices tested, improved sensitivity and full restoration of inclusivity for G1 viruses and has the potential to be used in different areas and contexts.

## 2. Materials and Methods

### 2.1. In Silico Update

The complete HA nucleotide sequences of avian and human H9Nx collected since 2015 in Eurasia and Africa were downloaded from the GISAID (Global Initiative on Sharing All Influenza Data) EpiFlu database [53] on 18 February 2021. The dataset includes H9Nx strains of the Y439, G1 and Y280 clades.

Sequences were aligned with MAFFT version 7 (Multiple Alignment with Fast Fourier Transform) [54,55] using default parameters. Geneious Prime 2020.1.2 (Biomatters Ltd., Auckland, New Zealand) [56] was employed to remove truncated or low quality sequences from the multisequence alignment (MSA) and to concatenate the hybridization regions of primers and probe developed by Monne and colleagues [48]. The concatenated-MSA (5338 records) was condensed to identify unique haplotypes (*n* = 480), and traced to assess the sequences composition within each haplotype using the SequenceTracer tool available on the Alignment Explorer web platform v1.4.3 (provided by the National Institute of Public Health, Czech Republic) [57,58]. A scoring system was adopted to categorize haplotypes based on their frequency and spatiotemporal distribution. Clusters of identical sequences with higher frequency, intercontinental spread and extended circulation over recent years were prioritized for primers and probe renewal. To assess the level of nucleotide variability at oligonucleotides binding regions within the concatenated-MSA, the Shannon entropy value *H*(i) was derived from the sequence logo generated with Geneious Prime 2020.1.2 (Biomatters Ltd., Auckland, New Zealand) [56]. Nucleotide positions with *H*(i) > 0.05 (corresponding to <1% sequences over the entire dataset) were carefully inspected for their polymorphisms. The occurrence of nucleotide signatures associated with priority haplotypes was adopted as a criterion for redesigning pan-H9 rRT-PCR oligonucleotides (Table 1). The IDT OligoAnalyzer online tool (Leuven, Belgium) [59] was employed to verify the physical properties of primers and probe.

After the completion of the validation pathway, assay inclusivity was verified in silico with newly released sequences (downloaded on 18 March 2022) using Geneious Prime 2020.1.2 (Biomatters Ltd., Auckland, New Zealand) [56].

### 2.2. rRT-PCR Assay Set Up

For the entire validation process, nucleic acids were isolated using the QIAsymphony DSP Virus/Pathogen Midi kit (Qiagen, Hilden, Germany) on a QIAsymphony SP instrument (Qiagen, Hilden, Germany) (sample volume 300 μL; custom protocol), unless otherwise specified. During the lysis phase, all the samples were spiked with an exogenous internal control (intype IC-RNA, Indical Bioscience GmbH, Leipzig, Germany) to reproduce routine laboratory conditions as foreseen by the upstream AIV screening method adopted at the IZSVe [60,61] that employs the same nucleic acids.

Amplification reaction was assembled with the AgPath-ID One-Step RT-PCR Reagents (Applied Biosystems, Waltham, MA, USA), 400 nM primer for, 200 nM each primer rev, 200 nM probe, 20 units RNase inhibitor and 5 μL template, in a final volume of 25 μL. Thermal cycling was performed on a CFX96 Deep Well Real-Time PCR System, C1000 Touch (Biorad, Hercules, CA, USA), as follows: 50 °C for 10 min, 95 °C for 10 min, followed by 45 cycles at 95 °C for 15 s, 54 °C for 30 s and 72 °C for 15 s. Data were analyzed using Bio-Rad CFX Manager software (Version 3.1) (Biorad, Hercules, CA, USA), with fluorescence drift correction for the baseline adjustment and single threshold manually set above the background noise (c.ca 50 RFU).

### 2.3. Analytical Specificity (Asp)

The capacity of the pan-H9 rRT-PCR to distinguish the target from other microorganisms and sample matrix components was assessed. In detail, exclusivity was verified by testing either AIV negative specimens from birds (swabs, organs homogenates) as well as avian nontarget bacteria and viruses, including different AIV HA subtypes. Inclusivity was confirmed on reference isolates available at the IZSVe repository of different origins and clades. Finally, selectivity was evaluated by testing different types of sample matrices, derived from confirmed H9 clinical cases. The complete list of samples tested for analytical specificity is available as Supplementary Materials (Table S1).

### 2.4. Analytical Sensitivity (ASe) and Repeatability

Tracheal and cloacal swabs rehydrated in PBSa, and oviduct homogenate (1:10 *w*/*v* in PBS with antibiotics and antimycotics—PBSa) collected from SFP chickens were contaminated with a dilution series of titrated H9 isolates of the Y439, G1 and Y280 clades. Each dilution was tested by rRT-PCR in triplicate. The highest dilution at which all the replicates tested positive identified the limit of detection (LoD). Intra- and interassay repeatability, expressed as percent coefficient of variation (%CV), was estimated in tracheal swabs and oviduct homogenates artificially contaminated with different doses of Y439 and G1 viruses (i.e., LoD, LoD + 1 log, LoD + 3 log) by two operators, on three different days.

For the pan-H9 rRT-PCR application as a frontline method for targeted diagnosis of this subtype, the possibility to coamplify the exogenous internal control in a duplex format was also explored in tracheal swabs and oviduct homogenates spiked with Y280.

To evaluate pan-H9 rRT-PCR performance under challenging conditions, serial dilutions of synthetic RNA (Ultramer RNA oligonucleotides, Integrated DNA Technologies, IDT, Leuven, Belgium) representing G1 and Y280 strains with multiple mismatches at the oligonucleotides binding regions, were also tested in triplicate. For comparison, a synthetic RNA with a perfect match (positive control) was also analyzed.

### 2.5. Diagnostic Sensitivity (DSe) and Specificity (DSp)

OIE guidelines [62] were followed to establish the number of field samples with known infectious status required to assess the diagnostic performances of the pan-H9 rRT-PCR, with the following estimates: 98% sensitivity and specificity, 5% error and 99% confidence. In total, 70 positive and 53 negative samples (including specimens positive for AIV subtypes other than H9) were tested (Table 2). More detailed information is available in Table S3. The AIV screening method in place at the IZSVe was run in parallel [60,61].

### 2.6. Reproducibility and Robustness

Two identical series of *n* = 13 oviduct homogenates, either negative or spiked with different loads of G1 or Y280 viruses, were analyzed in parallel with a territorial laboratory of the IZSVe (SCT1—Buttapietra, Verona). Nucleic acids were isolated with the MagMAX Pathogen RNA/DNA Kit (Applied Biosystems, Waltham, MA, USA) on a KingFisher Flex Processor (Thermo Fisher Scientific, Waltham, MA, USA) (sample volume 200 μL; protocol “low-cell-content samples”). For each sample aliquot, rRT-PCR was run in duplicate. Reproducibility was assessed also by participating in the OFFLU proficiency testing program 2021 for avian influenza A virus, H5 and H7 subtyping [63] organized by the Australian National Science Agency—Commonwealth Scientific and Industrial Research Organisation (CSIRO). Nucleic acids’s extraction was performed both with automatic and manual systems (i.e., MagMAX Pathogen RNA/DNA Kit, Applied Biosystems, Waltham, MA, USA and QIAamp Viral RNA Mini Kit, Qiagen, Hilden, Germany). Although H9 subtyping was not part of the assessable parameters of the exercise, the organization provided decoding for H9N2 samples included in the panel to allow self-assessment.

### 2.7. Procedural Modifications for Pan-H9 rRT-PCR Deployment

To facilitate assay deployment and circumvent possible issues deriving from reagents depletion, different kits for nucleic acids purification and amplification were evaluated.

#### 2.7.1. Automatic and Manual Nucleic Acids Isolation Kits

SPF tracheal and cloacal swabs rehydrated in PBSa and oviduct homogenate (1:10 *w*/*v* in PBSa) spiked with G1 were processed comparatively with: (i) QIAsymphony DSP Virus/Pathogen Midi kit (Qiagen, Hilden, Germany) as described above, (ii) MagMAX Pathogen RNA/DNA Kit (Applied Biosystems, Waltham, MA, USA) on a KingFisher Flex Processor (Thermo Fisher Scientific, Waltham, MA, USA) as described above, and (iii) NucleoSpin RNA (Macherey-Nagel, Dueren, Germany) (sample volume 100 μL; protocol “RNA purification from cultured cells and tissue”). Samples were analyzed in triplicate from the nucleic acids’ extraction phase. Amplification was carried out with the AgPath-ID One-Step RT-PCR Reagents (Applied Biosystems, Waltham, MA, USA), as previously detailed.

#### 2.7.2. One-Step Real-Time RT-PCR Kits

Nucleic acids isolated from tracheal and cloacal swabs (*n* = 24 positive samples) collected from G1 experimentally challenged chickens in the framework of a previous research project were used to evaluate the performance of different real-time RT-PCR kits. Amplification conditions were optimized for the QIAGEN OneStep RT-PCR Kit (Qiagen, Hilden, Germany) and the Quantitect Probe RT-PCR Kit (Qiagen, Hilden, Germany). Details of standardized protocols are available as Supplementary Materials. Performances were compared with the AgPath-ID One-Step RT-PCR Reagents (Applied Biosystems, Waltham, MA, USA), under the conditions described above.

### 2.8. Comparison with Existing Molecular Diagnostic Methods

A comprehensive literature review was carried out to identify available molecular methods for H9 diagnosis. rRT-PCR protocols fully validated and/or extensively used under field conditions, namely Saito et al. (2018) [52] and Hassan et al. (2019) [29] (later implemented in [64]), were selected to be compared with the new pan-H9 rRT-PCR and the original protocol by Monne et al. (2008) [48]. For this purpose, a subset of 46 H9-positive field samples employed for DSe were tested, applying the experimental conditions as per original papers. In parallel, the screening rRT-PCR targeting the AIV M-gene (Heine et al., 2015) [60,61] was also performed.

### 2.9. Statistical Analysis

Statistical analyses based on Ct values obtained for the (i) reproducibility, (ii) amplification kits comparison and (iii) evaluation of sensitivity for different real-time PCR assays (paragraphs 2.6, 2.7.2 and 2.8) were performed using Prism 9.3.1 (GraphPad, San Diego, CA, USA). Negative samples were arbitrarily assigned a value of 45. Student’s *t*-test (significance with *p* < 0.05) and Spearman’s rank correlation were estimated.

## 3. Results

### 3.1. Analytical Performance

#### 3.1.1. Exclusivity, Inclusivity and Selectivity

The capacity of the pan-H9 rRT-PCR to specifically detect only the target was confirmed upon verification of the analytical specificity. No background hybridization was recorded when testing negative tracheal/cloacal swabs and oviduct homogenates. The absence of cross-reaction was verified also against a wide panel of avian microorganisms, and exclusivity with respect to non-H9 AIV subtypes was also demonstrated. In contrast, the pan-H9 rRT-PCR successfully recognized a panel of H9N2, H9N6, H9N7 and H9N8 reference strains of different origins that tested positive at low Ct values (8 ≤ Ct ≤ 14.88). All represented clades (Y439, Y280, G1) were detected. The absence of inhibition and/or interference phenomena referable to the different types of sample matrix analyzed was preliminarily demonstrated with a selection of H9 clinical samples, which were successfully recognized in all cases. Detailed information is reported in Table S1.

#### 3.1.2. Analytical Sensitivity and Repeatability

In all cases but one, the LoD reached values close to 1 EID_50_/100 μL, independently of the virus clade and sample matrix combination tested. Only for Y280 in the oviduct, the pan-H9 rRT-PCR was 1 log less sensitive (Table 3). The method showed to be repeatable at all the dilutions tested, including the LoD, with an agreement between Ct values above 96% (Table S2).

Sensitivity tests were also run in duplex, coamplifying an exogenous internal control. Such experimental setting is required to assure the quality and conformity of analytical results and prevent issues deriving from an inhibition of the amplification reaction, in the event that the assay is employed as a frontline method for the targeted diagnosis of H9 viruses. Results for Y280 spiked into tracheal swabs and oviduct highlight that the LoDs of the simplex and duplex formats are the same, demonstrating that the coamplification of the internal control does not impair assay sensitivity (Table 3).

Finally, tolerance of the pan-H9 rRT-PCR to mismatches occurrence was evaluated by testing serial dilutions of synthetic RNAs representing G1 and Y280 viruses with different HA sequences. Overall, assay sensitivity was retained in the presence of two mismatches at the primers’ hybridization regions, yielding performance similar to the positive control with a perfect match. A more severe impact on the LoD and reaction efficiency was observed for synthetic RNAs with ≥6 mismatches (Table 4).

### 3.2. Diagnostic Performance with Clinical Samples

DSe and DSp were used as indicators to establish the capacity of the assay to correctly identify true positive and true negative field samples. In detail, H9-confirmed cases from a plethora of geographic origins, sample matrices and hosts were successfully detected with the pan-H9 rRT-PCR, with Ct values comparable to the AIV screening method targeting the M gene. No false positives were observed when testing H9-negative samples (Table S3). Overall, the diagnostic sensitivity and specificity values totaled 100%. Based on the Ct values yielded for H9-positive samples, a diagnostic cut-off of 35 is proposed.

### 3.3. Interlaboratory Reproducibility

The simultaneous testing of positive and negative oviduct homogenates at two different facilities of the IZSVe revealed a high reproducibility of the pan-H9 rRT-PCR, (*p* < 0.0001; r = 0.996) (Table S4) and is indicative of assay robustness. Only one sample corresponding to the LoD of G1 clade was missed by one of the two laboratories.

Additionally, all the H9 positive samples included in the OFFLU proficiency testing program 2021 were successfully identified, resulting in a perfect agreement between the pan-H9 rRT-PCR and the expected values (Cohen’s Kappa coefficient, K = 1).

### 3.4. Potential for Pan-H9 rRT-PCR Deployment

To facilitate the transferability of the pan-H9 rRT-PCR to third-party laboratories, the performance of different reagents was evaluated. First, three diverse nucleic acids purification systems were compared in terms of LoD, upon use of the same amplification kit (i.e., AgPath-ID One-Step RT-PCR Reagents, Applied Biosystems, Waltham, MA, USA). In all the sample matrices tested, the assay sensitivity was higher (3.16 EID_50_/100 μL) when using automatic extraction methods. In contrast, the NucleoSpin RNA (Macherey-Nagel, Dueren, Germany) appeared less efficient independently of the type of sample, with a LoD of 31.6 EID_50_/100 μL (Table 5).

**Figure 1:**
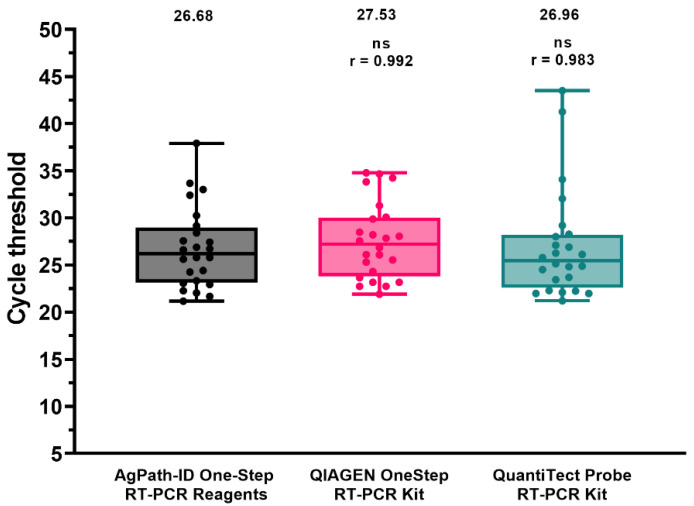
Performance comparison of different amplification kits. Dots represent individual Ct values. Mean Ct and Spearman’s rank correlation coefficient (r) are reported. ns: not significant.

Secondly, reaction conditions for two different amplification kits (i.e., the QIAGEN OneStep RT-PCR Kit and the Quantitect Probe RT-PCR Kit, Qiagen, Hilden, Germany) were optimized and compared to the standard protocol, using nucleic acids isolated from tracheal and cloacal swabs obtained from a previous H9 in vivo challenge (Figure 1). All the conditions allowed target detection and yielded similar Ct values with high correlation.

### 3.5. Comparison of the Pan-H9 rRT-PCRs with Other Assays

**Figure 2:**
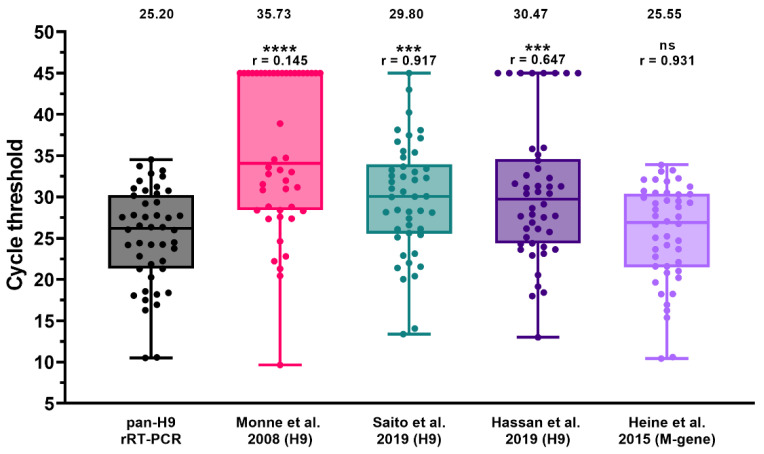
Diagnostic sensitivity of the pan-H9 rRT-PCR compared to M-gene screening rRT-PCR (Heine et al., 2015) [60] and three existing H9 detection methods [29,48,52]. Dots represent individual Ct values. Negative samples were arbitrarily assigned a value of 45. Mean Ct and Spearman’s rank correlation coefficient (r) are reported. **** *p* < 0.0001; *** *p* < 0.0002; ns: not significant.

A selection of field samples (*n* = 46) employed for the assessment of DSe was also used to compare the sensitivity of the pan-H9 rRT-PCR with respect to existing rRT-PCR methods for the identification of the H9 subtype, as well as for AIV screening (Figure 2 and Table S3). Overall, the pan-H9 rRT-PCR showed a significantly higher detection rate and sensitivity compared to other assays targeting this subtype. In detail, G1 viruses from Africa and the Middle East missed by the rRT-PCR by Monne et al. (2008) were successfully recognized with the new method. The assay developed by Saito et al. (2019) yielded one false negative and significantly higher Ct values (mean value: 28.80 vs. 25.20). Dropouts were observed also for the assay developed by Hassan and collaborators (2019), which was unable to recognize eight samples. For 13 Asian and African specimens comprising several G1-confirmed viruses, flat amplification plots and a low fluorescence were noticeable. When considering positive samples with sharp fluorescence curves, the differences in Ct values between the assay by Hassan et al. (2019) and the pan-H9 rRT-PCR were not statistically significant (r = 0.885; *p* = 0.538).

The pan-H9 rRT-PCR sensitivity was also compared with the screening rRT-PCR for AIV targeting the matrix (M) gene (Heine et al., 2015). All samples were identified by the new H9 assay, yielding Ct values comparable to that of the M-gene rRT-PCR (r = 0.931).

## 4. Discussion

The absence of notification obligations for H9Nx combined with insufficient surveillance for this subtype in countries with limited resources has led to an overall underestimation of its circulation [4,5]. This gap is worsened by the adverse effect of evolutionary drivers acting on viral diversification [41,65,66], which might result in diagnostic dropout for molecular-based methods. Nowadays, the resolution of such issues largely benefits from the constant increase of sequence data available that permits a timely update of diagnostic methods and improvement of laboratories responsiveness [67]. As a matter of fact, for clinical samples originating from Africa and the Middle East, we recently experienced failures in H9 identification by rRT-PCR [48] revealed by NGS analyses. This prompted us to undertake an in-depth update of the protocol originally designed by Monne and collaborators and widely used in different geographic areas and settings, with the aim of developing a pan-H9 rRT-PCR capable of recognizing the Y439, Y280 and G1 clades. To this end, a bioinformatics workflow was optimized to extract relevant data preparatory for the redesign of the oligonucleotides that account for the overall genetic variability of contemporary H9Nx. The new assay accommodates 95.3% of the entire dataset of sequences analyzed (5086 scores out of 5338) with ≤3 mismatches. The remaining sequences showed different degrees of variability at oligonucleotides binding regions and are mostly related to viral haplotypes that occurred only once, before 2018, and were extinct soon after. Although for the pan-H9 rRT-PCR design these sequences were considered negligible, assay inclusivity was verified in the presence of a high number of mismatches, confirming the potentiality of our method to identify even highly divergent viruses at high concentration, while avoiding unwanted cross-reaction with other AIV subtypes and common avian bacteria and viruses. In view of the frequent cocirculation of H9 viruses with other pathogens, assay specificity appears crucial in order to correctly identify disease etiology.

The newly designed pan-H9 rRT-PCR showed a high analytical sensitivity and repeatability in the sample matrices relevant for the tissue tropism of H9Nx viruses (i.e., tracheal and cloacal swabs and oviduct), reaching values between 1.51 and 14.12 EID_50_/100 μL for representative strains of the Y439, G1 and Y280 clades. Evaluation of diagnostic sensitivity was extended to a wider variety of sample matrices, hosts, geographic origins and viral haplotypes, and was assessed comparatively with other methods. In particular, in the routine practice, a comparison with Ct values obtained with AIV screening tests designed to maximize sensitivity and inclusivity represents a useful source of information for diagnosticians to infer semiquantitative data on the approximate viral load for specific AIV subtypes. In our settings, the pan-H9 rRT-PCR showed a performance comparable to that of the AIV M-gene assay [60,61], as assessed by a significant correlation in terms of Ct values. Thus, any remarkable discrepancy between H9 and M-gene assays should be critically evaluated, as it might be indicative of the emergence of mutant strains that possibly imply assays’ update. Notably, a comparison with existing H9 rRT-PCR protocols highlights that the new assay enables a significantly higher detection rate and lower Ct values, with restored capability to detect mutant viruses of the G1 clade. While limitations of the protocol by Monne and colleagues [48] were known for samples from Africa and the Middle East, drawbacks of the rRT-PCR by Hassan et al. [29] were unexpected due to its high inclusivity assessed in silico. A more careful analysis of false negatives and doubtful samples (i.e., flat amplification plots) from Benin and Nigeria for which sequence data are available, highlights the occurrence of one mismatch at the 9th position of the probe’s binding region. The same mismatch was previously found to be responsible for diagnostic dropouts that affected the assay developed by Monne and collaborators [48] and most likely could explain these outcomes as well.

One of the strategies of diagnostic laboratories to reduce costs while preserving results quality relies on the standardization of material, prioritizing the procurement of reagents already in use for routine activities and thus taking advantage of economies of scale. In order to facilitate this process for third-party laboratories willing to acquire the pan-H9 rRT-PCR, experimental conditions were optimized for different extraction systems and amplification kits. Automatic purification systems showed a better performance while reducing turnaround time and personnel occupation. Amplification conditions were optimized for different reagents from Thermo Fisher Scientific (Waltham, MA, USA) and Qiagen (Hilden, Germany), yielding comparable results. Chemistry dependence was observed for the TaqMan Fast Virus 1-Step Master Mix (Applied Biosystems, Waltham, MA, USA) and the SuperScript III One-Step RT-PCR System with Platinum *Taq* DNA Polymerase (Invitrogen, Waltham, MA, USA), both yielding higher Ct values and a lower sensitivity (data not shown). Because of their performance, these kits were not considered further in the validation process. Another important feature implemented in the pan-H9 rRT-PCR, is the inclusion of an exogenous internal control while maintaining the overall sensitivity. This option appears useful in case of a documented circulation of H9 viruses, for which targeted surveillance without prior M-gene screening is required to speed up viral detection and identification. These data confirm an overall high resilience of the pan-H9 assay to procedural modifications, as well as satisfactory reproducibility and robustness as also assessed by interlaboratory exercises carried out during the validation process.

## 5. Conclusions and Perspectives

Avian influenza viruses of the H9N2 subtype maintain the attention of animal health authorities high because of their growing prevalence and adverse impact on local poultry production, despite the extensive use of inactivated vaccines in endemic areas. Another important reason for H9 monitoring relies on its zoonotic potential, per se or secondary to the effect of reassortment with other AIV subtypes and pathotypes circulating in avian and mammalian species. The availability of sensitive and performant methods for H9 diagnosis is thus crucial for an early detection and to support surveillance activities aiming to improve animal health and prevent zoonotic events. The new real-time RT-PCR protocol for H9 detection was conceived to maximize its inclusivity and sensitivity, and was extensively validated and optimized for its application in different areas and settings. While this work aims to provide a reliable tool for H9 detection based on the most recent sequence data available, the genetic drift of AIV viruses, mostly involving the HA gene, imposes the periodical assessment of assay inclusivity with newly released sequences to verify whether the assay is still fit-for-purpose.

## Figures and Tables

**Table 1:** Primers and probe of the pan-H9 rRT-PCR assay. LNA-modified bases are in bold and underlined.

Oligonucleotide	Sequence 5′ → 3′	Nt. Position ^1^
Pan-H9 for	ATR GGG TTT GCT GCC	1615–1629
Pan-H9 rev1	TCA TAT ACA AAT GTT GCA YCT G	1662–1683
Pan-H9 rev2	TTA TAT ACA GAT GTT GCA YCT G	1662–1683
Pan-H9 probe	TTC TGG GCY A**T**G **T**CH AAY GG	1636–1655

^1^ Nucleotide position refers to strain A/pheasant/Italy/21VIR2284-1/2021 (GISAID reference number EPI1947247).

**Table 2:** Clinical samples with known infectious status used to assess DSe and DSp.

	Origin	Collection Year	Matrix	Species	No.
**H9-confirmed cases**	Europe	2018–2022	Swabs, stool	Mallard, teal, pheasant, goose, other unspecified avian species	22
	Africa	2019–2021	Swabs, organs, FTA	Chicken, cockerel, other unspecified avian species	21
	Middle East	2019–2021	FTA	Chicken	17
	Asia	2012–2021	Organs, FTA	Chicken	10
H9-negative samples *	Europe	2018–2021	Swabs, organs	Chicken, turkey, mallard, teal, pheasant, goose, quail, magpie, partridge, shoveler, duck, swan, gull	53

* Comprise H1, H3, H5 and H6 AIV positive samples.

**Table 3:** Analytical sensitivity in tracheal swabs, cloacal swabs and oviduct homogenate for H9 Y439, G1 and Y280 clades. The LoD is expressed as EID_50_/100 μL and mean Ct values. For each dilution series, the amplification efficiency *E* (%) and the coefficient of correlation R^2^ are reported. n.t.: not tested.

Strain	Tracheal Swab			Cloacal Swab			Oviduct		
	LoD (Ct)	*E* (%)	R^2^	LoD (Ct)	*E* (%)	R^2^	LoD (Ct)	*E* (%)	R^2^
A/pheasant/Italy/21VIR2284-22/2021/H9N2 (Y439 clade)	1.51 (33.85)	90.5	0.995	1.51 (33.48)	95.9	0.997	1.51 (34.80)	90.4	0.998
A/chicken/Nigeria/19VIR8424-15/2019/H9N2 (G1 clade)	3.16 (34.06)	99.9	0.996	3.16 (35.06)	94.8	0.998	3.16 (36.04)	99.4	0.998
A/chicken/Malaysia/2630-8/2012/H9N2 (Y280 clade)	1.41 (33.78)	95.6	0.998	1.41 (33.81)	98.0	0.997	14.12 (32.01)	97.8	0.996
A/chicken/Malaysia/2630-8/2012/H9N2 (Y280 clade) with intype IC-RNA *	1.41 (33.53)	97.2	0.998	n.t.	n.t.	n.t.	14.12 (32.13)	90.9	0.985

* rRT-PCR run in duplex format with the internal control.

**Table 4:** Analytical sensitivity of synthetic RNAs representing G1 and Y280 viruses. The LoD is expressed as copies/μL of RNA and mean Ct values. For each dilution series, the amplification efficiency *E* (%) and the coefficient of correlation R^2^ are reported.

Synthetic RNA *	No. of Mismatches			Performance		
	For	Probe	Revs	LoD (Ct)	*E* (%)	R^2^
MN038193 (G1)	2	0	0	10^1^ (38.98)	85.6	0.998
MK553893 (Y280)	0	1	6	10^5^ (37.56)	82.3	0.982
MN765147 (Y280)	0	0	2	10^2^ (34.54)	91.7	0.998
MN765086 (Y280)	0	1 (3′-end)	5	10^4^ (38.98)	78.5	0.973
Positive control ^§^	0	0	0	10^1^ (37.56)	86.4	0.993

* The GenBank accession number of the HA sequences used to produce synthetic RNAs is reported for each sample. ^§^ Synthetic RNA with perfect match with respect to primers and probe sequences.

**Table 5:** Analytical sensitivity in tracheal swabs, cloacal swabs and oviduct homogenate spiked with H9 G1 (A/chicken/Nigeria/19VIR8424-15/2019/H9N2) using different nucleic acids extraction kits. The LoD is expressed as EID_50_/100 μL and mean Ct values. For each dilution series, the amplification efficiency *E* (%) and the coefficient of correlation R^2^ are reported.

Nucleic Acids’ Purification Systems	Tracheal Swab			Cloacal Swab			Oviduct		
	LoD (Ct)	*E* (%)	R^2^	LoD (Ct)	*E* (%)	R^2^	LoD (Ct)	*E* (%)	R^2^
QIAsymphony DSP Virus/Pathogen Midi kit (Qiagen, Hilden, Germany)	3.16 (34.06)	99.9	0.996	3.16 (35.06)	94.8	0.998	3.16 (36.04)	99.4	0.998
MagMAX Pathogen RNA/DNA Kit (Applied Biosystems, Waltham, MA, USA)	3.16 (35.29)	93.8	0.993	3.16 (33.97)	98.7	0.999	3.16 (34.87)	98.8	0.994
NucleoSpin RNA (Macherey-Nagel, Dueren, Germany)	31.6 (35.47)	104.9	0.995	31.6 (34.38)	95.4	0.997	31.6 (34.68)	94.6	0.992

## Data Availability

The data presented in this study are reported in the main text or are available as Supplementary Materials.

## Supplementary Materials

The following supporting information can be downloaded at: https://www.mdpi.com/article/10.3390/v14061263/s1, Table S1: Analytical specificity; Table S2: Within-run and between-days repeatability; Table S3: Diagnostic sensitivity and specificity and assays comparison; Table S4: Reproducibility; File S1: Amplification conditions with different real-time PCR kits.
