# Supplementary material for: Redesign and Validation of a Real-Time RT-PCR to Improve Surveillance for Avian Influenza Viruses of the H9 Subtype
Source: Viruses. 2022 Jun 10;14(6):1263. doi: 10.3390/v14061263 (PMC9227555; doi:10.3390/v14061263)
Supplement: Supplementary file 1 [file viruses-14-01263-s001.zip › viruses-1713850-supplementary File S1.pdf]

pan-H9 rRT-PCR amplification conditions with different real-time PCR kits

1. QIAGEN OneStep RT-PCR Kit (Qiagen)

Amplification reaction was assembled with 400 nM primer for, 200 nM each primer rev, 200 nM probe, 4 units RNase inhibitor, 3 mM $MgCl_2$ and 5 µl template, in a final volume of 25 µl. Thermal cycling was performed on a CFX 96 Deep well Real-Time PCR System, C1000 Touch (Biorad), as follows: 50°C for 30 min, 95°C for 15 min, followed by 45 cycles at 94°C for 10 sec, 54°C for 35 sec and 72°C for 10 sec. Data were analyzed using the Bio-Rad CFX Manager software (Version 3.1), with fluorescence drift correction for baseline adjustment and single threshold manually set above the background noise (c.ca 50 RFU).

2. Quantitect Probe RT-PCR Kit (Qiagen)

Amplification reaction was assembled with 400 nM primer for, 200 nM each primer rev, 200 nM probe, 20 units RNase inhibitor, 1.5 mM $MgCl_2$ and 5 µl template, in a final volume of 25 µl. Thermal cycling was performed on a CFX 96 Deep well Real-Time PCR System, C1000 Touch (Biorad), as follows: 50°C for 30 min, 95°C for 15 min, followed by 45 cycles at 94°C for 15 sec, 54°C for 60 sec and 72°C for 10 sec. Data were analyzed using the Bio-Rad CFX Manager software (Version 3.1), with fluorescence drift correction for baseline adjustment and single threshold manually set above the background noise (c.ca 50 RFU).
